# Earlier breeding, lower success: does the spatial scale of climatic conditions matter in a migratory passerine bird?

**DOI:** 10.1002/ece3.1824

**Published:** 2015-11-19

**Authors:** Annegret Grimm, Brigitte M. Weiß, Lars Kulik, Jean‐Baptiste Mihoub, Roger Mundry, Ulrich Köppen, Tomas Brueckmann, Ruth Thomsen, Anja Widdig

**Affiliations:** ^1^ Behavioral Ecology Research GroupInstitute of BiologyUniversity of LeipzigLeipzigGermany; ^2^ Department of Conservation BiologyUFZ‐Helmholtz Centre for Environmental ResearchLeipzigGermany; ^3^ Junior Research Group of Primate Kin SelectionDepartment of PrimatologyMax Planck Institute for Evolutionary AnthropologyLeipzigGermany; ^4^ Max Planck Institute for Evolutionary AnthropologyLeipzigGermany; ^5^ Beringungszentrale HiddenseeGreifswaldGermany; ^6^ Grüne LIGA Kohrener LandKohren‐SahlisGermany; ^7^ Department of AnthropologyUniversity College LondonLondonUK; ^8^ German Center for Integrative Biodiversity Research (iDiv)LeipzigGermany

**Keywords:** Barn swallow, breeding success, climate change, *Hirundo rustica*, local temperature, NAO, North Atlantic Oscillation, phenology, scale sensitivity

## Abstract

Following over 20 years of research on the climatic effects on biodiversity we now have strong evidence that climate change affects phenology, fitness, and distribution ranges of different taxa, including birds. Bird phenology likely responds to changes in local weather. It is also affected by climatic year‐to‐year variations on larger scales. Although such scale‐related effects are common in ecology, most studies analyzing the effects of climate change were accomplished using climatic information on a single spatial scale. In this study, we aimed at determining the scale‐dependent sensitivity of breeding phenology and success to climate change in a migratory passerine bird, the barn swallow (*Hirundo rustica*). For both annual broods, we investigated effects of local weather (local scale) and the North Atlantic Oscillation (NAO, large scale) on the timing of breeding and breeding success. Consistent with previous studies in migratory birds we found that barn swallows in Eastern Germany bred progressively earlier. At the same time, they showed reduced breeding success over time in response to recent climatic changes. Responses to climatic variation were observed on both local and large climatic scales, but they differed with respect to the ecological process considered. Specifically, we found that the timing of breeding was primarily influenced by large‐scale NAO variations and to a lesser extent by local weather on the breeding grounds. Conversely, climatic conditions on the local scale affected breeding success, exclusively. The observed decrease in breeding success over years is likely a consequence of scale‐related mismatches between climatic conditions during different breeding phases. This provides further evidence that a species' response of earlier breeding may not be enough to cope with climate change. Our results emphasize the importance of considering the response of ecological processes along different climatic scales in order to better understand the complexity of climate change effects on biodiversity.

## Introduction

Since the mid‐20th century the earth's climate has warmed in an unprecedented manner, with anthropogenic drivers like greenhouse gas emission being the dominant cause of the observed warming (IPCC 2014). This has raised concerns about whether and how species and populations can cope with changing climatic conditions. In order to predict the ecological consequences of climate change, we need a thorough understanding of whether and how individuals respond and how populations are affected by contemporary variations in climatic variables (Weatherhead 2005).

Changes in the phenology (i.e., periodicity of life cycles) of living organisms are among the best‐documented responses to climatic conditions and have been observed across all taxa and diverse environments (reviewed or meta‐analyzed, e.g., in Walther et al. 2002; Parmesan and Yohe 2003). In particular, many bird species have advanced their spring migration and onset of breeding over the last decades (Crick et al. 1997; Rubolini et al. 2007; Charmantier et al. 2008). There is evidence that this shift is causally related to increasing spring temperatures (Forchhammer et al. 1998; McCleery and Perrins 1998; Both et al. 2004). In addition to such linear, long‐term trends, several studies emphasized that bird phenology also responds to short‐term, year‐to‐year variation in the environment, concordant with large‐scale climate phenomena (e.g., the North Atlantic Oscillation [NAO] or the El Niño Southern Oscillation [ENSO], Forchhammer et al. 1998, 2002; Przybylo et al. 2000; Walther et al. 2002; Møller 2002; Hüppop and Hüppop 2003; Stenseth et al. 2003; Weatherhead 2005).

Responses to climatic conditions may vary considerably between species and populations (Visser et al. 2003; Both et al. 2004; Rubolini et al. 2007). Parmesan and Yohe (2003) showed that of the 168 bird species, 78 species advanced and 14 species delayed their breeding onset. Birds may profit from these phenological adjustments, as individuals breeding early often have larger clutch sizes or higher overall reproductive success than those breeding later (e.g., Hatchwell 1991; Winkler et al. 2002). Indeed, measures of breeding success were positively related to temperatures on the breeding grounds in several European and North American birds (Barnagaud et al. 2011; Mihoub et al. 2012; Van Oudenhove et al. 2014). However, increasingly warmer springs do not correspond to enhanced reproductive success in all bird species and populations (e.g., Winkler et al. 2002; Ludwig et al. 2006).

Climatic variation occurs at different spatial scales, which can blur our understanding of the response of species to climate change. Although climatic conditions are often correlated across scales, large‐scale climatic indices were frequently found to better predict ecological processes compared to local weather measures (Hallett et al. 2004; Weatherhead 2005). On the other hand, several studies demonstrated that reproductive parameters and nestling condition were related to local temperature or rainfall (e.g., Keller and Van Noordwijk 1994; Dickey et al. 2008), indicating that local weather variation may also affect reproductive success. Only few studies assessed the role of the different scales simultaneously (e.g., Hüppop and Hüppop 2003; Hallett et al. 2004; Weatherhead 2005; Dickey et al. 2008). Hence, understanding scale‐dependent patterns in responses to climate change remains a major challenge but may contribute to understanding the capacity of species and populations to adapt to a changing environment.

Adjusting breeding behavior to climatic fluctuations at different scales may be particularly challenging for long‐distance migratory birds, as they experience multiple habitats and climatic regimes throughout the year that may require different adaptations (Forchhammer et al. 2002). Furthermore, timing of long‐distance migration is triggered by cues that are only partly related to the local conditions on the breeding grounds. If migration and breeding are influenced by different environmental cues and along different scales, scale‐dependent responses are thus unlikely to be optimal on all scales simultaneously. Disentangling the relative contribution of the different ecological factors and associated scales shaping responses to climate change is therefore of crucial importance.

The barn swallow (*Hirundo rustica*) is a widespread and well‐studied long‐distance migratory bird that is abundant but has been reported as declining across Europe over the past 20 years (e.g., Møller 2004). Recent studies showed that breeding phenology and brood size of Danish colonial barn swallows were affected in a complex manner by either the NAO (Møller 2002) or ecological processes on a small geographical scale (Møller 2008), highlighting the importance of considering the impact of different climatic scales. The present study investigates the effects of local‐ and large‐scale climatic variation on the timing of breeding and breeding success of barn swallows in Eastern Germany. Specifically, we aim at (1) identifying influences of climatic variation on the timing of breeding and breeding success of first and second broods and (2) disentangling effects of small‐scale (local) versus large‐scale (regional) climatic conditions. For this purpose we tested whether timing of breeding and breeding success of barn swallows in Eastern Germany changed between 1997 and 2010, and whether these changes mirrored climatic variation on different scales. We considered monthly temperature and precipitation in the study area as representatives of local‐scale climatic variation and the NAO index as a measure of large‐scale, regional climatic variation. In particular, we expected the NAO to be a better indicator for the timing of breeding given that it affects large parts of the Northern Hemisphere and has the potential to be informative for migratory birds already before arrival on the breeding grounds. Conversely, we expected breeding success to be better predicted by immediate, local weather conditions during brood rearing.

## Materials and Methods

### Data

#### Study species data

**Figure 1:**
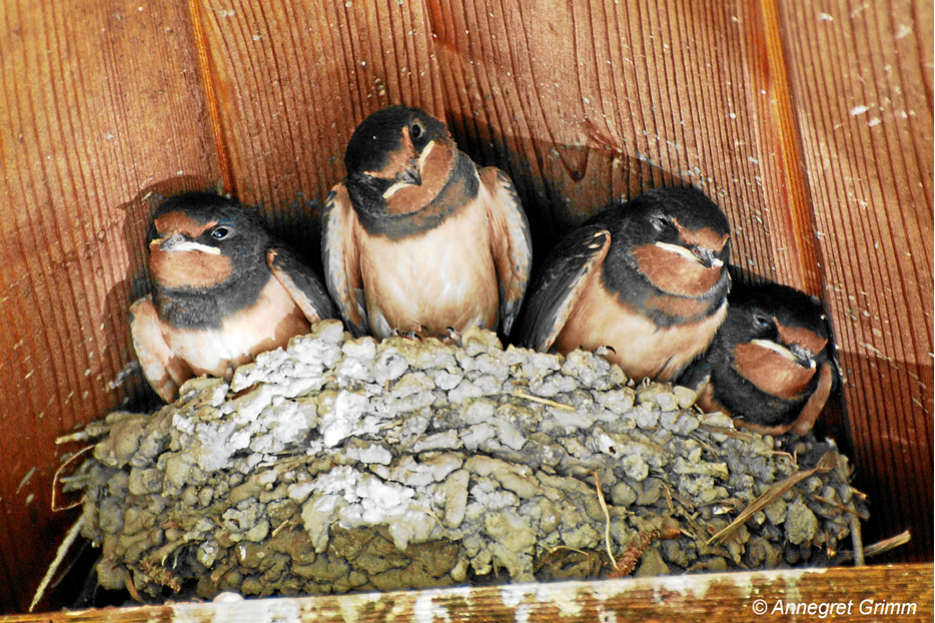
A clutch of barn swallow (*Hirundo rustica*) fledglings.

**Figure 2:**
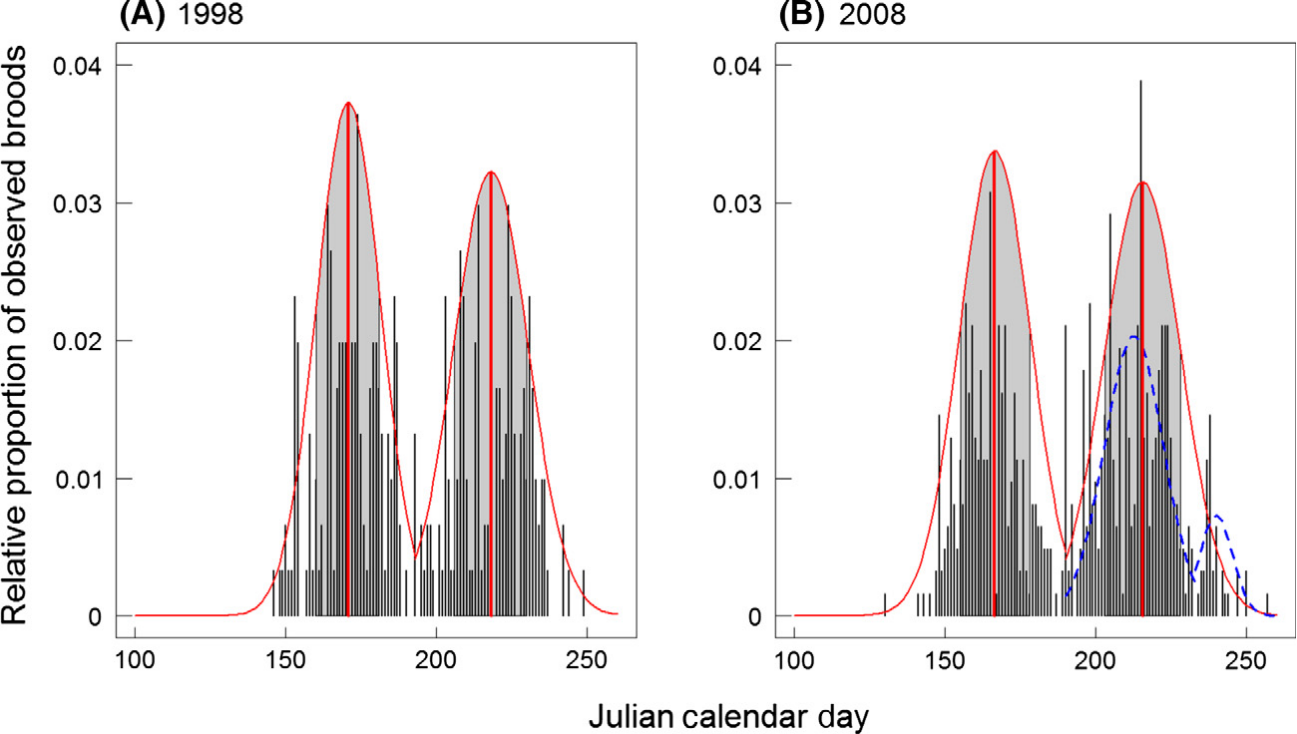
Number of observed broods per Julian Calendar Day for two representative years: 1998 (A) and 2008 (B). The red solid lines show a normal distribution and its mean for the first and second broods (number of broods per day) in the respective year. The gray shaded area includes 68.27% of all observed broods per season (standard deviation of the normal distributions). The blue dashed line in 2008 symbolizes an emerging bimodal pattern of the second brood over years, potentially reflecting a third brood peak.

Barn swallows are small, semicolonial, insectivorous, migrant passerine birds breeding all over Europe (Fig. 1). They are widespread and abundant but show decreasing population trends (Inger et al. 2015). Barn swallows are socially monogamous and are most frequently double‐brooded, with a first clutch following the arrival from wintering quarters in spring and a second clutch later in summer (see Fig. 2; Møller 1989, 2002). Incubation lasts approximately 14 days and hatchlings are fed by both parents up to 20 days (Møller 1989). Breeding populations from Germany and Eastern Europe migrate back to their wintering grounds in Southern Africa (Hobson et al. 2012, 17 ring recoveries from our study area) between August and October.

We used data from annual ringing of barn swallows across Eastern Germany (six federal states: Mecklenburg, Brandenburg, Berlin, Thuringia, Saxony, and Saxony‐Anhalt) collected and provided by the Hiddensee Bird Ringing Centre (Hiddensee Ringing Data Communication no. 17/2014). Ringing of barn swallows in the study region began in 1964, but we only considered records from 1997 to 2010 due to the lack of consistent data in previous years. Since 1997, ringing was conducted within an Eastern Germany ringing framework where certain places where visited every year and all reachable hatchlings of appropriate size (see below) were ringed (U. Köppen, own observation). Recorded information included ring ID of the hatchlings, brood size (i.e., number of hatchlings per nest), ringing date, and exact nest location (determined by GPS). If this information was incomplete, we removed the respective data point from the dataset. As almost no information about the parents' ID was available, we could not assign broods to parents or second broods to the corresponding first broods.

The time window for ringing hatchlings is very short because chicks must be old enough to have tarsi of sufficient length, while older chicks (15–20 days) might already be able to fly and can leave the nest when they are disturbed (Geiter and Bairlein 2001). Thus, chicks were ringed approximately at the same age, that is, between 10 and 15 days. As a result, the ringing date is a reliable proxy of the overall timing of breeding, reflecting the timing of arrival, laying, and hatching. Due to a very low nestling mortality in barn swallows (<1%, Møller 2002), we considered brood size at the time of ringing as a reliable proxy of the breeding success of breeding pairs in our analyses. The sampling unit in our analyses was the brood (i.e., specified through a specific date, location, and number of hatchlings) rather than the individual. The distinction between first and second broods was made by means of the annual distributions of ringing dates (Fig. 2). Overall, we used 7256 broods over 14 years (first brood: *N* = 3754; second brood: *N* = 3502).

#### Climate data

Temperature (°C) and rainfall (mm) were used as proxies for variability in local, small‐scale climatic conditions (i.e., weather). Weather data were obtained from the German Weather Service (Deutscher Wetterdienst, http://www.dwd.de). We used monthly mean temperatures and precipitation averaged across the federal states of Eastern Germany representing our study region. Local climatic variables potentially influencing the timing of breeding of barn swallows were considered to occur immediately before or in the early beginning of breeding. As barn swallows typically arrive on their European breeding grounds in April (Møller 1989), we considered weather conditions in April as relevant for the timing of breeding of the first brood. Since first broods are completed by late June or early July (Table S3), we considered weather conditions in July as relevant for the timing of the subsequent second brood. Regarding breeding success, we assumed brood size is a product of successive breeding phases from laying, incubation, and hatching up to fledgling survival. As a consequence, the weather from early stages of breeding to parental care needed to be considered. We therefore used weather conditions in April and May for the first brood, and July and August for the second brood. During our study period, temperatures in April and July were generally increasing (0.09 and 0.07°C per year, respectively), while temperatures in May and August showed a decreasing temporal trend (−0.10 and −0.08°C per year, respectively). Likewise, precipitation in April was decreasing (−1.00 mm per year), whereas rainfall in May, July, and August was increasing (2.05 mm, 2.15 mm, and 1.90 mm per year, respectively).

The large‐scale regional climatic conditions of the northern hemisphere are dominated by atmospheric oscillations over the North Atlantic between the Subtropics and the Arctic, which are summarized in the North Atlantic Oscillation (NAO) index. This index reflects the most reliable climate pattern in the northern hemisphere and is frequently used as a “climate package” (Stenseth et al. 2003; Barnagaud et al. 2011). In Eastern Germany, positive NAO indices are related to warmer and wetter weather conditions, while negative indices reflect colder and drier conditions (Hurrell 1995; Stenseth et al. 2003). Monthly NAO indices as proxies for regional climatic conditions of the northern hemisphere were provided by the Climate Prediction Center, National Centers for Environmental Prediction, National Oceanic and Atmospheric Administration, USA (ftp://ftp.cpc.ncep.noaa.gov/cwlinks/). We used the annual NAO index averaging all monthly values to reflect the long‐term nature of the phenomenon and because annual NAO indices were found to predict local climatic conditions both in spring and in summer (Møller 2002).

### Statistical analyses

To investigate changes in the timing of breeding and breeding success over time as well as their potential relations to climatic conditions, we used generalized linear mixed models (GLMM, McCullagh and Nelder 1989; Baayen 2008; Bolker et al. 2009). We performed our analyses in R 3.1.1 (R Core Team 2014) using the packages *lme4* (Bates et al. 2014) and *MCMCglmm* (Hadfield 2010) as well as *AICcmodavg* (Mazerolle 2014), *MuMIn* (Barton 2015), *hier*.*part* (Walsh and Mac Nally 2013), and *gplots* (Warnes et al. 2014). Importantly, analyses for first and second broods were conducted separately because we were not able to reliably control for their possible pseudoreplication effects emerging from correlations of successive broods within a pair due to unknown identities of the parents.

We first tested for temporal trends in the timing of breeding (response variable: ringing date in Julian days) and breeding success (response variable: brood size) by considering year as a single fixed effects test predictor in any of the two broods. For analyses of breeding success, we further tested for a combination of between‐year and within‐year variation in the timing of breeding by considering both year and Julian day as fixed effects test predictors.

Second, we investigated whether potential temporal variations in the timing of breeding and breeding success were related to climatic conditions at different scales. In models investigating small‐scale effects, local temperatures, rainfall, and their interactions were included as fixed effects test predictors substituting the fixed effect of year. Since possible influences on both the timing of breeding and breeding success are not necessarily limited to local scales (e.g., Stenseth et al. 2003), we further built models using the annual NAO index as a large‐scale climatic test predictor testing both linear and quadratic relationships consistent with Møller (2002). We included both relationships as we assumed that either breeding conditions might become optimal toward one side of the NAO index (linear relation) or optimal breeding conditions might be around an NAO of 0 and might get worse both above and below 0 (quadratic relation). Effects of local weather and regional climate were first assessed in separate models because climatic conditions at small (local) and large (regional) scales are frequently correlated and could thus violate model assumptions (Trigo et al. 2002; Stenseth et al. 2003).

All fixed effect test predictors (temperatures, precipitations, and NAO indices) were *z*‐transformed to a mean of zero and a standard deviation of one. Additional to fixed effects test predictors we included control predictors in all models (Mundry 2014). Control predictors were not relevant with respect to our hypotheses but have known effects that needed to be controlled for to allow valid conclusions about our test predictors. In models investigating the timing of breeding, we included latitude as a fixed effects control predictor and year and locality as random effects, including random slopes of latitude within years (Schielzeth and Forstmeier 2009; Barr et al. 2013). In analyses investigating breeding success, latitude and Julian day standardized to a minimum of 1 per year were included as fixed effects control predictors and year and locality as random effects; we further included latitude and Julian day as random slopes within years. Random effects were included to control for pseudoreplication as part of the experimental design. Their variances can be found in Appendix S5. We did not include random slopes within locality in any of the models as most locality points occurred just once. A comprehensive overview describing all predictors as well as further model details including tests of model assumptions are provided in Appendix S1.

To assess the influence of individual parameters we fitted all possible models that could be built out of a given set of test predictors as we did not have any a priori hypotheses on the subsets of test predictors in question (Stephens et al. 2007). We compared Gaussian models (models on the timing of breeding) using Akaike's information criterion (AIC; Burnham and Anderson 2002; Johnson and Omland 2004) and zero‐truncated Poisson models (models on breeding success) using the deviance information criterion (DIC; Spiegelhalter et al. 2002) (see S1 for model details). Statistical differences between models were considered when differences between their AIC/DIC scores were larger than 2. The relative importance of parameters in GLMMs was calculated by summing up AIC/DIC weights (*ω*AIC/*ω*DIC) for each variable across models. Parameter estimates and standard errors were obtained as model averaged estimates by means of multimodel inference (Burnham and Anderson 2002), and their *P*‐values with LRTs of the full model against the model without the effect in question. Although GLMM comparisons were conducted with *z*‐transformed temperature and precipitation values, coefficient estimates presented in the results were obtained using original data.

Third, we assessed the relative influence of small‐ versus large‐scale climatic conditions on the timing of breeding and breeding success using two approaches. In a first approach, we ran across‐scale GLMMs that included statistically relevant small‐ and large‐scale climatic variables determined by the previous analyses as test predictors and all control predictors (see Appendix S1). With this across‐scale approach we aimed at determining the weighted influences of all test predictors. In a second approach, the statistically relevant small‐ and large‐scale climatic variables were subjected to hierarchical variance partitioning (Lee and Nelder 1996; Mac Nally 2002) that calculated model fits according to all possible combinations of explanatory variables. Here, hierarchical modeling explicitly depicts scale dependency (Hartel et al. 2010). Thus, this method disentangles the independent contribution of all fixed effects as a fraction of total variation explained, and joint effects that are equally well explained by any variable. In both approaches, analyses for the timing of breeding and breeding success as well as for first and second broods were conducted separately.

In addition to the mixed model analyses and hierarchical partitioning, we compiled breeding data into yearly measures with the according sample size of 13 (one per year of observation excluding 2010 [insufficient data]). In particular, we determined the minimum, maximum, and standard deviation of the timing of breeding (in Julian days) for the first and the second broods. We also calculated the total duration of the breeding season per year as the number of days between the first and the last record of each year. The mean annual breeding productivity was calculated by summing brood sizes of the first and the second broods divided by the total number of broods for each year. We conducted Pearson's correlations to test for temporal trends in these measures within our study period. When a temporal trend was found, pairwise correlations were conducted with each local and regional climatic variable described earlier.

## Results

### Timing of breeding

**Figure 3:**
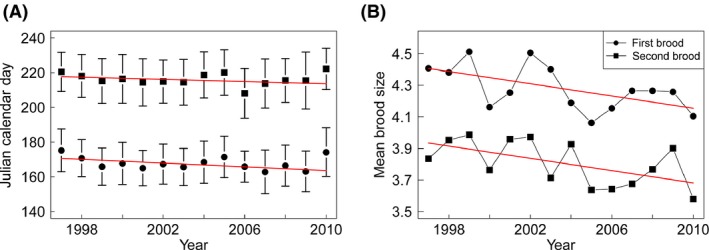
Timing of breeding (A) as mean Julian Calendar Day and breeding success (B) as mean brood size of both broods between 1997 and 2010. Standard deviations are represented with bars (A). Red lines illustrate a significant decrease.

A negative temporal trend in the timing of breeding demonstrated earlier timing for both first and second broods (first brood: *P* = 0.0073; second brood: *P* < 0.001; Fig. 3). However, the annual change in timing was slightly higher for the first (−0.55 ± 0.19 days) than for the second brood (−0.37 ± 0.23 days) although that difference was not tested for significance.

**Figure 4:**
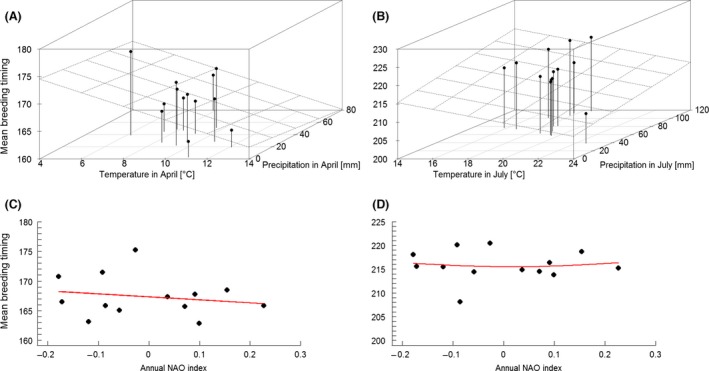
Timing of breeding (as mean Julian Calendar Day) of first (A, C) and second (B, D) broods in relation to local (temperature, rainfall, and their interaction; A, B) and regional (annual NAO index; C, D) climatic drivers. The plane (A, B) represents the modeled relation of the timing of breeding to temperature and precipitation. Solid red lines (C, D) show temporal trends of the fitted models. While statistical analyses are based on single brood observations, this graph was produced using mean annual values.

For the first brood, the highest weighted small‐scale climatic driver was temperature in April followed by precipitation in April with the model with the lowest AIC including the interaction between them (Tables 1 and S2.1). However, the difference to the model including only temperature in April was just 0.32 AIC points, whereas differences to all other models were >2 AIC points (Table S2.1). Both higher temperatures and rainfall were associated with earlier breeding (Fig. 4). Moreover, a linear trend with annual NAO indices as a large‐scale climatic driver was observed, with higher NAO indices leading to earlier breeding (Table 1, Fig. 4).

**Table 1:** Estimates of all test predictors affecting the timing of breeding and breeding success of first broods. GLMMs were conducted separately for local (T: temperature, P: precipitation) and regional (NAO) climatic factors (see Materials and Methods and Appendix S1). *P*‐values for single terms were derived from a model without interaction if the interaction was not significant

Test predictor	Timing of breeding				Breeding success			
	Estimate	Standard error	*P*‐value	Σ(*ω*AIC)	Estimate	Standard error	*P*‐value	Σ(*ω*DIC)
T (April)	−1.99	0.53	a	0.81	−0.00448	0.002	0.63	0.05
P (April)	−0.42	0.06	a	0.56	−0.0007	0.004	0.13	0.12
T:P (April)	0.07	0.03	0.0405	0.37	0.00005	0.00003	0.08	0.03
T (May)	–	–	–	–	0.0083	0.002	0.002	0.97
P (May)	–	–	–	–	−0.0006	0.0006	0.16	0.13
T:P (May)	–	–	–	–	0.0003	0.0001	0.22	0.04
NAO (linear)	−1.51	0.85	0.0699	0.48	0.01	0.03	<0.001	0.78

a *P*‐value not indicated because it is conditional on another predictor and thus does not have a meaningful interpretation (Aiken and West 1991; Schielzeth 2010).

When comparing the relative influence of small‐ versus large‐scale climatic conditions affecting the timing of breeding, temperature in April was the highest weighted parameter (*ω*AIC = 0.92), followed by precipitation in April (*ω*AIC = 0.82), and the annual NAO index (*ω*AIC = 0.75). The best model included all three parameters (Appendix S4). Hierarchical partitioning showed that the annual NAO index had the largest independent effect (33.88% variance explained), followed by temperature (17.91%) and precipitation in April (2.36%).

For the second brood, the highest weighted small‐scale climatic effect was precipitation in July followed by temperature in July, with the best model including the interaction between them (Tables 2 and S2.2). Again, higher temperature and rainfall led to earlier breeding (Fig. 4). In contrast to the first brood, the large‐scale models including annual NAO indices were not statistically different from the null model but the quadratic relationship was more supported (Table 2, Fig. 4). However, the NAO was the highest weighted parameter (*ω*AIC = 0.99) across scales, followed by temperature (*ω*AIC = 0.96) and precipitation in July (*ω*AIC = 0.86), with the best model including all three parameters (Appendix S4). Hierarchical partitioning showed comparable results, with the annual NAO index explaining most of the variance (29.55%), followed by precipitation (21.81%) and temperature in July (14.31%).

**Table 2:** Estimates of all test predictors affecting the timing of breeding and breeding success of second broods. GLMMs were conducted separately for local (T: temperature, P: precipitation) and regional (NAO) climatic factors (see Materials and Methods and Appendix S1)

Test variable	Timing of breeding				Breeding success			
	Estimate	Standard error	*P*‐value	Σ(*ω*AIC)	Estimate	Standard error	*P*‐value	Σ(*ω*DIC)
T (July)	−1.77	0.42	a	0.67	−0.01	0.002	a	0.46
P (July)	−0.19	0.03	a	0.85	−0.001	0.0002	a	0.44
T:P (July)	0.03	0.02	0.0264	0.48	0.0002	0.0001	<0.005	0.05
T (August)	–	–	–	–	0.004	0.001	a	0.90
P (August)	–	–	–	–	−0.004	0.0001	a	0.79
T:P (August)	–	–	–	–	0.0006	0.0003	<0.005	0.64
NAO (linear)	−70.04	51.54	0.1462	0.32	0.98	0.23	<0.005	0.09
NAO (quadratic)	67.25	50.43		0.33	1.15	0.36		0.37

a *P*‐value not indicated because it is conditional on another predictor and thus does not have a meaningful interpretation (Aiken and West 1991; Schielzeth 2010).

In addition to effects on individual broods we determined changes in yearly metrics of the timing of breeding (Table S3, see exemplarily Fig. 2). The minimum date of breeding became significantly earlier over the course of the study period for first, but not for second broods (first brood: *t*_11_ = −3.57, *r* = −0.732, *P* = 0.004; second brood: *t*_11_ = −1.57, *r* = −0.43, *P* = 0.14). The same was true for the mean date of breeding (first brood: *t*_11_ = −2.52, *r* = −0.606, *P* = 0.028; second brood: *t*_11_ = −1.41, *r* = −0.39, *P* = 0.19), while no significant relationship was observed for the maximum date of breeding (first brood: *t*_11_ = −0.90, *r* = −0.26, *P* = 0.39). However, the timing of second broods became significantly more variable (i.e., showed a larger standard deviation) over time (*t*_11_ = 2.89, *r* = 0.656, *P* = 0.015). The total duration of the breeding season also increased significantly over time (*t*_11_ = 3.48, *r* = 0.723, *P* = 0.005). Furthermore, the standard deviation in second broods was significantly higher in years with higher April temperatures (*t*_11_ = 3.93, *r* = 0.784, *P* = 0.002). We detected no other relationships between yearly breeding measures and climatic variables (all *P* > 0.05).

### Breeding success

Breeding success in the first and second broods tended to decrease over the study period (first brood: *P* = 0.05; second brood: *P* = 0.05; Fig. 3). The annual decline was similar in both broods (first brood: −0.0042 ± 0.0022 hatchlings; second brood: −0.0046 ± 0.0024 hatchlings). Accordingly, the annual breeding productivity also decreased over time (Pearson's correlation, *t*_12_ = −2.64, *r* = −0.607, *P* = 0.021; Fig. S3). The breeding success of both broods was also influenced by within‐year changes in the timing of breeding, with later broods showing decreased brood sizes in both breeding cycles (first brood: −0.0046 ± 0.0006, *P* < 0.001; second brood: −0.0045 ± 0.0006, *P* < 0.001).

**Figure 5:**
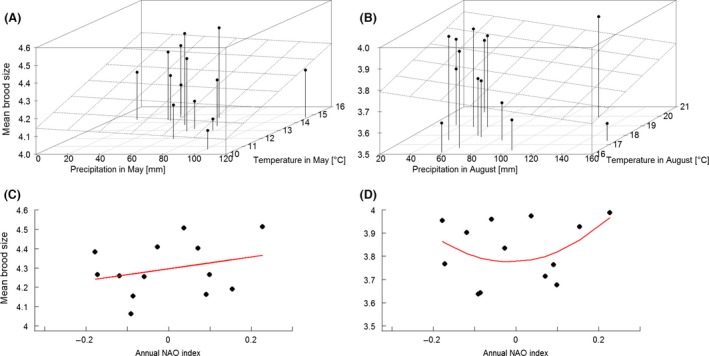
Breeding success (as mean annual brood size) of first (A, C) and second (B, D) broods in relation to local (temperature, rainfall, and their interaction; A, B) and regional (annual NAO index; C, D) climatic drivers. The plane (A, B) represents the modeled relation of the timing of breeding to precipitation and temperature. Solid red lines (C, D) show temporal trends of the fitted models. While statistical analyses are based on single brood observations, this graph was produced using mean annual values.

Brood size of first broods was influenced by small‐scale climatic variables, whereby the model with the lowest DIC contained only temperature in May (Tables 1 and S2.3, Fig. 5). Temperature in May also showed the highest weight (*ω*DIC = 0.97). The highest weighted large‐scale climatic variable was the linear NAO (Table 1, Fig. 5). In the across‐scale analysis, temperature in May was weighted highest (*ω*DIC = 0.83) while the annual NAO index showed the lowest (*ω*DIC = 0.18). The best model included temperature in May, exclusively (Appendix 4). In hierarchical partitioning temperature in May explained more variance (53.50%) than the annual NAO index (22.24%).

Brood size of second broods seemed to be mostly driven by local temperature and precipitation in August, with the best model including the interaction between them (Tables 2 and S2.4). In particular, higher temperatures and less rainfall led to larger broods (Fig. 5). The best large‐scale climatic model appeared to be the null model. However, the quadratic NAO model was not statistically different from the null model (Table 2, Fig. 5). Across‐scale analyses showed that precipitation in August had the highest weight (*ω*DIC = 0.98), followed by temperature in August (*ω*DIC = 0.92) and the annual NAO index (*ω*DIC = 0.81). The best model included all three parameters (Appendix 4). Hierarchical partitioning indicated that precipitation in August explained most of the variance in brood sizes (32.87%), followed by temperature in August (24.87%) and the annual NAO index (13.42%).

## Discussion

The results of the present study strengthen existing evidence that climatic conditions affect the phenology and breeding success of birds. Breeding success of barn swallows in Eastern Germany is negatively affected by recent climate changes. As in many other bird species studied to date (e.g., Crick et al. 1997; Forchhammer et al. 1998; Both et al. 2004), barn swallows bred earlier in warmer years and, consistent with temporal temperature trends, increasingly earlier over the study period. At the same time, reproductive success declined for both broods, corresponding to the population declines observed in barn swallows in recent decades (Inger et al. 2015).

Relationships between climatic and breeding parameters have been described on local (e.g., Charmantier et al. 2008; Caro et al. 2009; Mihoub et al. 2012) and large spatial scales (e.g., Forchhammer et al. 1998; Przybylo et al. 2000; Barnagaud et al. 2011), but only few studies assessed the role of the different scales simultaneously (e.g., Hüppop and Hüppop 2003; Hallett et al. 2004; Weatherhead 2005; Dickey et al. 2008). We observed responses of breeding barn swallows to climate on two spatial scales, but the relative contribution of each spatial scale exhibited contrasting patterns for the timing of breeding and breeding success. Consistent with our initial hypotheses, the timing of breeding was predominantly affected by large‐scale conditions. While the NAO index and local weather conditions were weighted equally, the NAO explained a larger proportion of variance in the timing of breeding. Both local and regional weather parameters affected breeding success, with local parameters being rated as more important than regional ones in the across‐scale analyses. Likewise, Hüppop and Hüppop (2003) found that the timing of spring migration in European birds was related to both local temperatures en route and the NAO, with the effects of NAO being more apparent. Comparable to our results in a trans‐Saharan migrant species, the Arctic migratory greater snow geese (*Chen caerulescens atlantica*) also showed a strong dependency to local climatic variations in addition to a large‐scale climatic phenomenon (Dickey et al. 2008). Together with results of these previous studies our findings emphasize that responses to climatic variations need to be investigated on different climatic scales, as scale dependency may greatly vary with respect to the ecological parameters under investigation. Aggregating more evidences from other organisms, regions, or biological systems is critical to get more general conclusions and to better understand the complexity of scale‐dependent responses to climate change.

Neglecting spatial scales could further lead to deceptive results, as demonstrated in this study where (1) local weather appeared to affect the timing of breeding and (2) regional climate affected breeding success in single scale analyses. However, across‐scale analyses showed that the contribution of local weather to the timing of breeding was outweighed by that of the regional NAO index. Only for the timing of first broods did local weather has higher AIC weights than the NAO index, indicating that effects of local weather may be relatively robust and present in many of the models. Our hierarchical partitioning approach, however, showed that the NAO index still explained the largest proportion of variance. Likewise, across‐scale analyses showed that local climate is more important for breeding success and explained more variance (see Appendix 4). Thereby, our results also demonstrate strong limitations of single scale analyses. Although parameters on one single scale might appear to have direct effects on timing and success of breeding, their total effect might be diminished across scales.

Over the course of our study, barn swallows of Eastern Germany bred progressively earlier. This advancement of breeding was stronger for first than for second broods and led to an overall increase in the duration of the breeding season. Similarly, the breeding interval between first and second broods had become longer in a Danish population of barn swallows in recent decades (Møller 2007). In our study population, the phenological distribution of second broods further became bimodal from 2006 onward (see exemplarily Fig. 2), suggesting that the breeding season had become long enough to accommodate a possible third brood. If third broods indeed become a regular phenomenon in this population, they might offer a potential way to mitigate the negative effects of the overall reduced brood sizes (see below).

Mean timing of breeding did not just advance, it also responded to yearly climatic conditions and in particular to fluctuations of the NAO and temperatures in April. Adjusting the timing of breeding to yearly variation in climatic conditions on the breeding grounds poses a particular challenge for long‐distance migrants. Their breeding phenology is to some extent constrained by spring arrival on the breeding grounds and thus by the ability to adjust the timing of migration en route or even from their wintering grounds (Forchhammer et al. 2002). Nonetheless, some migratory species, including barn swallows, appear to be capable of such an adjustment because they were found to migrate earlier in years with warmer spring temperatures at breeding grounds or stop‐over sites (Huin and Sparks 1998; Forchhammer et al. 2002; Hüppop and Hüppop 2003). Large‐scale climatic phenomena such as the NAO may serve as regional indicators that provide information about climatic conditions on the breeding grounds well before arrival. Indeed, previous research indicated that a climatic connectivity between African wintering grounds and European breeding grounds might allow migratory birds to use cues in wintering areas to predict meteorological conditions in breeding areas and to adjust their migration schedules to optimize arrival dates (Saino and Ambrosini 2008). Along with reducing the interval between arrival and breeding, earlier arrival may thus enable long‐distance migrants to breed earlier in warmer springs (e.g., Forchhammer et al. 2002; Both and te Marvelde 2007). While possible effects of the NAO on arrival dates and, consequently, on the timing of breeding are intuitive for first broods, the timing of second broods could also be adjusted using cues at the breeding grounds. However, the timing of second broods is linked to that of first broods (Smith et al. 1987; Møller 2007) which may explain why the NAO index also explained the largest proportion of variance in the timing of second broods in our study population.

Earlier breeding has been shown to be advantageous in numerous species (Verhulst and Tinbergen 1991; Rowe et al. 1994; Siikamäki 1998). Consistent with these findings the present study showed that early broods were generally larger than late broods. Brood size was also positively related to local climatic variables, in particular to temperatures during late breeding and during the brood rearing period. These patterns insinuate that barn swallows can cope with or even profit from recent climate changes, however this was not the case in our study. Brood sizes successively decreased over time, suggesting that adjusting the timing of breeding to spring temperatures was not an adequate response for maintaining breeding success. A possible explanation might be that climatic trends differ during the different breeding phases. Specifically, local temperatures prior to and during the early incubation period (i.e., April for first, July for second broods) increased over the study period, while temperatures during late stages of incubation and brood rearing (i.e., May for first, August for second broods) decreased. Such a climatic mismatch is comparable to previous findings in Finnish black grouse (*Tetrao tetrix*), where spring, but not summer temperatures, had increased, leading to earlier breeding but increased chick mortality due to hatching when climatic conditions were suboptimal (Ludwig et al. 2006).

Temperature and precipitation may affect hatchlings directly or indirectly, for example, through food availability. Barn swallows rely on insects for feeding their offspring (Møller 2011) and matching the timing of breeding with local food availability is of critical importance for insectivorous birds (e.g., Siikamäki 1998; Burger et al. 2012). However, the rate of phenological change may differ considerably depending on the trophic level of a given species (see Both et al. 2009; Thackeray et al. 2010). Insufficient advancement of laying date relative to the phenological advancement of prey species has been associated with reduced fledging success in various insectivorous bird species (e.g., Burger et al. 2012; Reed et al. 2013). Climate‐related food availability in early life may even have long‐term consequences, as it was found to affect the likelihood of fledglings to recruit into the breeding population and their future reproductive success (Reed et al. 2013; Herfindal et al. 2015). Declining brood sizes in barn swallows in our study area thus may be related to weather‐mediated availability of flying insects and associated nestling survival.

Responses to climate change may vary considerably between species and even between populations (e.g., great and blue tits: Visser et al. 2003; flycatchers: Both et al. 2004). We found that barn swallows in Eastern Germany partly differed in their responses to climatic conditions from those previously reported for Danish barn swallows (Møller 2002). In contrast to our study, Møller (2002) found no temporal trends for either the timing of breeding (measured as laying date) or brood size despite a comparable increase in April temperatures over time in both populations. However, Mihoub et al. (2012) suggested that the relative distribution of a population within the species' range may influence how temperatures affect breeding behavior; in the case of barn swallows, the Danish population might likely experience a coastal temperate climate contrasting with the rather continental climate in Eastern Germany, which results in smaller temperature differences in Denmark. This might explain why the timing of breeding was affected by variation in the NAO index in the Eastern German population (this study) but not in the Danish population (Møller 2002). Alternatively, the different time periods covered by the two studies (1997–2010 vs. 1970–2000, respectively) may be associated with different NAO trends. In particular, the NAO index was generally more often positive during Møller's study period but typically close to zero or negative and without any particular temporal trend in the present study. Accordingly, the positive NAO values experienced by Danish barn swallows may have favored a timing of breeding that was close to the behavioral or physiological limit for early breeding, while the Eastern German population experienced lower and more variable NAO values that may have provided more room for adjusting the timing of breeding.

In contrast to the timing of breeding, breeding success was barely impacted by large‐scale climatic variation in both populations (Møller 2002, this study). Rather, brood size was affected by climatic conditions on a local scale in the present study, highlighting that the relative effects of climatic conditions on ecological patterns might be strongly scale‐dependent. This brings two important implications for assessing population and species responses to climatic conditions. First, when comparing findings of different studies, the scales at which environmental or climatic conditions were considered are of critical importance, since comparisons between populations and/or species may be meaningless if conclusions are drawn along different scales. Second, a better understanding of the scale dependency of different ecological processes is required in order to produce more accurate predictions of population and the response of species to climate change. Accordingly, more studies are needed that address the effects of climatic variation on demography and life history along different spatial climatic scales.

The present study also has implications with respect to biodiversity monitoring. Limited monitoring effort was spent to sample data that now contributed to understanding the ecological consequences of climate change along different spatial scales. Such data, however, have significant limitations for understanding details such as which breeding stages were affected by climatic variation. Furthermore, we could not relate individual breeding parameters of second broods to those of the respective first broods nor assess between‐year individual variation because we do not know the identity of the parental birds. Nevertheless, our study demonstrates the usefulness of low‐effort monitoring data because their wide availability for different species over long time periods makes them particularly valuable for studying questions related to long‐term phenomena like climate change.

To conclude, we found that barn swallows in Eastern Germany bred progressively earlier but showed reduced breeding success in response to recent climate changes. This indicates that responding to increasing temperatures with earlier breeding may not be sufficient to cope with climate change. Importantly, responses to climatic variation were observed on both local and large climatic scales. The timing of breeding was primarily influenced by large‐scale NAO variations, while reduced breeding success appeared to be the consequence of local‐scale climatic mismatches during different breeding phases. Hence, the present study highlights the importance of considering different climatic scales for studying responses to climate change and in particular for understanding species and population differences therein.

## Data Accessibility

All data are available online or on request from the following institutions: Bird ringing data: Hiddensee Bird Ringing Centre (Hiddensee Ringing Data Communication no. 17/2014). German weather data: Deutscher Wetterdienst (http://www.dwd.de/bvbw/appmanager/bvbw/dwdwwwDesktop?_nfpb=true&_pageLabel=_dwdwww_klima_umwelt_klimadaten_deutschland&activePage=&_nfls=false). NAO index: Climate Prediction Center, National Centers for Environmental Prediction, National Oceanic and Atmospheric Administration, USA (ftp://ftp.cpc.ncep.noaa.gov/cwlinks/).

## Conflict of Interest

None declared.

## Supporting information


**Appendix S1** Overview of predictors used in the GLMMs and model details.

**Appendix S2** Model comparison tables of all local models.

**Appendix S3** Metrics of timing of breeding and productivity.

**Appendix S4** Model comparison tables and results for combined local and regional drivers.

**Appendix S5** Estimates of random effects control predictors.
