# Supplementary material for: Earlier breeding, lower success: does the spatial scale of climatic conditions matter in a migratory passerine bird?
Source: Ecol Evol. 2015 Nov 19;5(23):5722–34. doi: 10.1002/ece3.1824 (PMC4813123; doi:10.1002/ece3.1824)
Supplement: Supplementary file 1 — Appendix S1 Overview of predictors used in the GLMMs and model details. [file ECE3-5-5722-s001.docx]

**Supporting Information 1. Overview of predictors used in the GLMMs and model details**

## Predictors used in the GLMMs

A detailed and comprehensive overview describing all test and control predictors considered throughout the models built for the timing of breeding and breeding success is provided in Table S1.To assess the impact of not modelling random slopes for locality we ran an additional model into which we included random slopes for locality using a reduced dataset that exclusively included localities occurring at least three times. As the coefficients and standard errors of the respective fixed effects were similar for the full and reduced data set (results not shown), we present results based on the full data set.

Table S1. Detailed overview describing all test and control predictors considered throughout the models built for the timing of breeding and breeding success. T=temperature; P=precipitation; ”:” symbolises interactions; number represent the month of the year.

|  | | Fixed effects test predictors | | | | | | | | | | | | Fixed effects control predictors | | Random effects control predictors | | Random slope control predictors |
| --- | --- | --- | --- | --- | --- | --- | --- | --- | --- | --- | --- | --- | --- | --- | --- | --- | --- | --- |
|  |  | T(4) | P(4) | T:P(4) | T(5) | P(5) | T:P(5) | T(7) | P(7) | T:P(7) | T(8) | P(8) | T:P(8) | Latitude | Julian day | Year | Locality | by-year |
| Timing of Breeding | 1^st^ brood | x | x | x |  |  |  |  |  |  |  |  |  | x |  | x | x | latitude |
|  | 2^nd^ brood |  |  |  |  |  |  | x | x | x |  |  |  | x |  | x | x | latitude |
| Breeding success | 1^st^ brood | x | x | x | x | x | x |  |  |  |  |  |  | x | x | x | x | latitude and Julian day |
|  | 2^nd^ brood |  |  |  |  |  |  | x | x | x | x | x | x | x | x | x | x | latitude and Julian day |

## Model details

For all analyses determining changes in the timing of breeding (response: time in Julian days), we conducted LMMs (i.e., Gaussian error distribution and identity link function). Visual inspections of residual plots of all Gaussian models did not reveal any obvious deviations from homoscedasticity or normality assumption.

Analyses of breeding success (response: count data of brood size) were first checked by means of a GLMM with Poisson error distribution and log link function. All Poisson models were underdispersed (dispersion parameters between 0.31 and 0.35) due to the fact that there were no zeros in the count data. Thus, we used a zero-truncated Poisson error distribution as implemented in the R-package *MCMCglmm* (Hadfield 2010).

We evaluated significance of the full models with Likelihood Ratio Tests (LRT) comparing the full models (i.e., including all test and control predictors) with the respective null models (i.e., excluding test predictors, Forstmeier & Schielzeth, 2011; Mundry, 2011). All models were statistically different from the null models unless otherwise explicitly stated.

## References

Forstmeier W, Schielzeth H (2011) Cryptic multiple hypotheses testing in linear models: overestimated effect sizes and the winner’s curse. Behavioral Ecology and Sociobiology, **65**, 47–55.

Hadfield JD (2010). MCMC Methods for Multi-Response Generalized Linear Mixed Models: The MCMCglmm R Package. Journal of Statistical Software, **33**, 1-22. URL http://www.jstatsoft.org/v33/i02/.

Mundry R (2011) Issues in information theory-based statistical inference - a commentary from a frequentist’s perspective. Behavioral Ecology and Sociobiology, **65**, 57–68.
