# Supplementary material for: Earlier breeding, lower success: does the spatial scale of climatic conditions matter in a migratory passerine bird?
Source: Ecol Evol. 2015 Nov 19;5(23):5722–34. doi: 10.1002/ece3.1824 (PMC4813123; doi:10.1002/ece3.1824)
Supplement: Supplementary file 2 — Appendix S2 Model comparison tables of all local models. [file ECE3-5-5722-s002.docx]

**Supporting Information 2. Model comparison tables of all local models**

## Timing of Breeding

Table S2.1. Model comparison for local drivers influencing the timing of breeding of the first clutch. 1 refers to the intercept, *z* refers to z-transformed values, *T* and *P* refer to temperature and precipitation, respectively, and the number behind them represents the month of the year. The number of parameters includes all control parameters and the residual standard deviation (Appendix S1).

| **model** | **number of parameters** | **AIC** | **ΔAIC** | **AIC weight** | **cumulative weight** | **log likelihood** |
| --- | --- | --- | --- | --- | --- | --- |
| 1+z.T4+z.P4+z.T4:z.P4 | 9 | 26001.21 | 0 | 0.37 | 0.37 | -12991.61 |
| 1+z.T4 | 7 | 26001.53 | 0.32 | 0.32 | 0.69 | -12993.76 |
| 1+z.T4+z.P4 | 8 | 26003.41 | 2.20 | 0.12 | 0.81 | -12993.70 |
| 1 | 6 | 26003.43 | 2.22 | 0.12 | 0.93 | -12995.71 |
| 1+z.P4 | 7 | 26004.68 | 3.47 | 0.07 | 1 | -12995.34 |

Table S2.2. Model comparison for local drivers influencing the timing of breeding of the second clutch. 1 refers to the intercept, *z* refers to z-transformed values, *T* and *P* refer to temperature and precipitation, respectively, and the number behind them represents the month of the year. The number of parameters includes all control parameters and the residual standard deviation (Appendix S1).

| **model** | **number of parameters** | **AIC** | **ΔAIC** | **AIC weight** | **cumulative weight** | **log likelihood** |
| --- | --- | --- | --- | --- | --- | --- |
| 1+z.T7+z.P7+z.T7:z.P7 | 9 | 28063.70 | 0 | 0.48 | 0.48 | -14022.85 |
| 1+z.P7 | 7 | 28064.88 | 1.18 | 0.26 | 0.74 | -14025.44 |
| 1+z.T7+z.P7 | 8 | 28066.63 | 2.93 | 0.11 | 0.85 | -14025.31 |
| 1+z.T7 | 7 | 28067.18 | 3.48 | 0.08 | 0.93 | -14026.59 |
| 1 | 6 | 28067.65 | 3.96 | 0.07 | 1 | -14027.83 |

## Breeding success

Table S2.3. Model comparison for local drivers influencing breeding success of the first clutch. 1 refers to the intercept, *z* refers to z-transformed values, *T* and *P* refer to temperature and precipitation, respectively, and the number behind them represents the month of the year. The number of parameters includes all control parameters and the residual standard deviation (Appendix S1).

| **model** | **number of parameters** | **DIC** | **ΔDIC** | **DIC weight** | **cumulative weight** | **log likelihood** |
| --- | --- | --- | --- | --- | --- | --- |
| 1+z.T5 | 7 | 13618.25 | 0.00 | 0.857 | 0.857 | -6806.46 |
| 1+z.P4+z.T5+z.P5 | 9 | 13623.86 | 5.61 | 0.052 | 0.910 | -6810.84 |
| 1+z.P4+z.T5+z.P5+z.T5:z.P5 | 10 | 13624.7 | 6.45 | 0.034 | 0.943 | -6809.19 |
| 1+z.T4+z.P4+z.P5+z.T4:z.P4 | 10 | 13626.61 | 8.36 | 0.013 | 0.956 | -6807.50 |
| 1+z.T4+z.T5+z.P5 | 9 | 13626.78 | 8.53 | 0.012 | 0.968 | -6811.59 |
| 1+z.P5 | 7 | 13628.24 | 9.99 | 0.006 | 0.975 | -6812.22 |
| 1+z.T4+z.P4+z.T5+z.P5+z.T4:z.P4 | 11 | 13628.25 | 10.00 | 0.006 | 0.980 | -6811.06 |
| 1+z.T4+z.P4+z.T4:z.P4 | 9 | 13629.03 | 10.78 | 0.004 | 0.984 | -6811.68 |
| 1+z.T4 | 7 | 13629.73 | 11.48 | 0.003 | 0.987 | -6812.02 |
| 1+z.T4+z.P4+z.T5+z.P5 | 10 | 13629.74 | 11.49 | 0.003 | 0.989 | -6812.79 |
| 1+z.T4+z.P4+z.T5+z.P5+z.T4:z.P4+z.T5:z.P5 | 12 | 13630.04 | 11.79 | 0.002 | 0.992 | -6811.06 |
| 1+z.T4+z.P4+z.P5 | 9 | 13630.12 | 11.87 | 0.002 | 0.995 | -6811.95 |
| 1+z.T5+z.P5+z.T5:z.P5 | 9 | 13630.61 | 12.36 | 0.002 | 0.996 | -6812.40 |
| 1+z.T5+z.P5 | 8 | 13631.09 | 12.84 | 0.001 | 0.997 | -6810.08 |
| 1+z.T4+z.P4+z.T5+z.P5+z.T5:z.P5 | 11 | 13631.79 | 13.54 | 0.001 | 0.998 | -6811.88 |
| 1+z.T4+z.P4+z.T5+z.T4:z.P4 | 10 | 13632.3 | 14.05 | 0.001 | 0.999 | -6813.52 |
| 1 | 6 | 13634.12 | 15.87 | 0.000 | 0.999 | -6816.23 |
| 1+z.P4+z.P5 | 8 | 13634.17 | 15.92 | 0.000 | 1 | -6813.52 |
| 1+z.T4+z.T5+z.P5+z.T5:z.P5 | 10 | 13634.45 | 16.20 | 0.000 | 1 | -6811.22 |
| 1+z.T4+z.T5 | 8 | 13635.46 | 17.21 | 0.000 | 1 | -6812.00 |
| 1+z.T4+z.P5 | 8 | 13636.85 | 18.60 | 0.000 | 1 | -6812.35 |
| 1+z.T4+z.P4 | 8 | 13637.42 | 19.17 | 0.000 | 1 | -6811.71 |
| 1+z.T4+z.P4+z.T5 | 9 | 13637.58 | 19.33 | 0.000 | 1 | -6811.69 |
| 1+z.P4 | 7 | 13643.31 | 25.06 | 0.000 | 1 | -6814.00 |
| 1+z.P4+z.T5 | 8 | 13647.88 | 29.63 | 0.000 | 1 | -6812.97 |

Table S2.4. Model comparison for local drivers influencing breeding success of the second clutch. 1 refers to the intercept, *z* refers to z-transformed values, *T* and *P* refer to temperature and precipitation, respectively, and the number behind them represents the month of the year. The number of parameters includes all control parameters and the residual standard deviation (Appendix S1).

| **model** | **number of parameters** | **DIC** | **ΔDIC** | **DIC weight** | **cumulative weight** | **log likelihood** |
| --- | --- | --- | --- | --- | --- | --- |
| 1+z.T8+z.P8+z.T8:z.P8 | 9 | 11315.63 | 0.00 | 0.857 | 0.857 | -6806.46 |
| 1+z.P7+z.T8+z.P8+z.T8:z.P8 | 10 | 11316.10 | 0.47 | 0.052 | 0.909 | -6810.84 |
| 1+z.T7+z.T8+z.P8+z.T8:z.P8 | 10 | 11316.74 | 1.11 | 0.034 | 0.943 | -6809.19 |
| 1+z.T7+z.T8 | 8 | 11317.59 | 1.96 | 0.013 | 0.956 | -6807.50 |
| 1+z.T7+z.P7+z.T8+z.P8 | 10 | 11317.98 | 2.35 | 0.012 | 0.968 | -6811.59 |
| 1+z.T7+z.P7+z.T8+z.P8+z.T8:z.P8 | 11 | 11318.78 | 3.15 | 0.006 | 0.974 | -6812.22 |
| 1+z.T7+z.P8 | 8 | 11319.36 | 3.73 | 0.006 | 0.980 | -6811.06 |
| 1+z.P7+z.T8 | 8 | 11319.37 | 3.74 | 0.004 | 0.983 | -6811.68 |
| 1+z.P7 | 7 | 11320.22 | 4.59 | 0.003 | 0.986 | -6812.02 |
| 1+z.T8 | 7 | 11320.37 | 4.74 | 0.003 | 0.989 | -6812.79 |
| 1+z.T7+z.P7+z.P8+z.T7:z.P7 | 10 | 11320.80 | 5.17 | 0.002 | 0.992 | -6811.06 |
| 1+z.T7+z.P7+z.T8+z.T7:z.P7 | 10 | 11321.58 | 5.95 | 0.002 | 0.994 | -6811.95 |
| 1+z.T7 | 7 | 11322.51 | 6.88 | 0.002 | 0.996 | -6812.40 |
| 1+z.T7+z.P7+z.T8+z.P8+z.T7:z.P7 | 11 | 11322.83 | 7.20 | 0.001 | 0.997 | -6810.08 |
| 1+z.T7+z.P7+z.T7:z.P7 | 9 | 11323.68 | 8.05 | 0.001 | 0.998 | -6811.88 |
| 1+z.T7+z.P7 | 8 | 11323.83 | 8.20 | 0.001 | 0.999 | -6813.52 |
| 1+z.T7+z.P7+z.T8+z.P8+z.T7:z.P7+z.T8:z.P8 | 12 | 11324.88 | 9.25 | 0.000 | 0.999 | -6816.23 |
| 1+z.P7+z.T8+z.P8 | 9 | 11324.99 | 9.36 | 0.000 | 0.999 | -6813.52 |
| 1+z.T8+z.P8 | 8 | 11325.13 | 9.50 | 0.000 | 1 | -6811.22 |
| 1+z.P7+z.P8 | 8 | 11326.25 | 10.62 | 0.000 | 1 | -6812.00 |
| 1+z.T7+z.T8+z.P8 | 9 | 11326.96 | 11.33 | 0.000 | 1 | -6812.35 |
| 1 | 6 | 11327.54 | 11.91 | 0.000 | 1 | -6811.71 |
| 1+z.T7+z.P7+z.T8 | 9 | 11328.63 | 13.00 | 0.000 | 1 | -6811.69 |
| 1+z.P8 | 7 | 11329.74 | 14.11 | 0.000 | 1 | -6814.00 |
| 1+z.T7+z.P7+z.P8 | 9 | 11330.57 | 14.94 | 0.000 | 1 | -6812.97 |
