# Supplementary material for: Earlier breeding, lower success: does the spatial scale of climatic conditions matter in a migratory passerine bird?
Source: Ecol Evol. 2015 Nov 19;5(23):5722–34. doi: 10.1002/ece3.1824 (PMC4813123; doi:10.1002/ece3.1824)
Supplement: Supplementary file 3 — Appendix S3 Metrics of timing of breeding and productivity. [file ECE3-5-5722-s003.docx]

**Supporting Information 3. Metrics of the timing of breeding and productivity**

Table S3. Metrics of the timing of breeding (number of observed broods per day, see Fig. 1) for first and second broods. Min: Minimum, Max: Maximum, Sd: Standard deviation.

| Year | First brood | | | | Second brood | | | | total duration |
| --- | --- | --- | --- | --- | --- | --- | --- | --- | --- |
|  | Min | Mean | Max | Sd | Min | Mean | Max | Sd |  |
| 1997 | 151 | 175.25 | 199 | 12.32 | 205 | 220.47 | 250 | 11.28 | 99 |
| 1998 | 146 | 170.77 | 188 | 10.71 | 193 | 218.07 | 249 | 12.36 | 103 |
| 1999 | 145 | 165.88 | 189 | 10.75 | 192 | 215.24 | 250 | 12.93 | 105 |
| 2000 | 136 | 167.89 | 191 | 12.18 | 192 | 216.40 | 250 | 14.03 | 114 |
| 2001 | 134 | 165.04 | 187 | 10.21 | 189 | 214.45 | 246 | 13.62 | 112 |
| 2002 | 141 | 167.33 | 194 | 11.55 | 198 | 214.89 | 257 | 12.46 | 116 |
| 2003 | 141 | 165.67 | 187 | 10.72 | 189 | 214.50 | 253 | 13.18 | 112 |
| 2004 | 138 | 168.46 | 193 | 12.15 | 194 | 218.69 | 253 | 13.29 | 115 |
| 2005 | 142 | 171.46 | 197 | 11.98 | 198 | 220.07 | 250 | 13.11 | 108 |
| 2006 | 138 | 165.88 | 182 | 8.93 | 184 | 208.12 | 247 | 14.30 | 109 |
| 2007 | 137 | 162.84 | 190 | 12.61 | 191 | 213.78 | 249 | 14.18 | 112 |
| 2008 | 130 | 166.45 | 192 | 11.81 | 194 | 215.52 | 257 | 12.66 | 127 |
| 2009 | 134 | 163.13 | 187 | 11.75 | 189 | 215.47 | 253 | 16.39 | 119 |

Figure S3. Annual breeding productivity (sum of brood sizes of first and second broods divided by the total number of broods for each year). The solid red line shows a significant decrease over years (Pearson’s correlation, t_12_ = -2.64, r = -0.607, p = 0.021; slope estimate = -0.02, standard error = 0.007).
