# Supplementary material for: Earlier breeding, lower success: does the spatial scale of climatic conditions matter in a migratory passerine bird?
Source: Ecol Evol. 2015 Nov 19;5(23):5722–34. doi: 10.1002/ece3.1824 (PMC4813123; doi:10.1002/ece3.1824)
Supplement: Supplementary file 4 — Appendix S4 Model comparison tables and results for combined local and regional drivers. [file ECE3-5-5722-s004.docx]

**Supporting Information 4. Model comparison tables and results for combined local and regional drivers**

## First clutch

Table S4.1. Model comparison for local and regional drivers influencing the timing of breeding of the first clutch. 1 refers to the intercept, *z* refers to z-transformed values, *T* and *P* refer to temperature and precipitation, respectively, and the number behind them represents the month of the year. The number of parameters includes all control parameters and the residual standard deviation (Appendix S1).

| **model** | **number of parameters** | **AIC** | **ΔAIC** | **AIC weight** | **cumulative weight** | **log likelihood** |
| --- | --- | --- | --- | --- | --- | --- |
| 1+z.T4+z.P4+NAO+z.T4:z.P4 | 10 | 29055.19 | 0 | 1 | 0.561 | -14517.59 |
| 1+z.T4+z.P4+z.T4:z.P4 | 9 | 29057.84 | 2.65 | 0.266 | 0.710 | -14519.92 |
| 1+z.T4+NAO | 8 | 29059.09 | 3.90 | 0.142 | 0.790 | -14521.54 |
| 1+z.T4+z.P4+NAO | 9 | 29059.72 | 4.53 | 0.104 | 0.848 | -14520.86 |
| 1+z.T4 | 7 | 29060.31 | 5.12 | 0.077 | 0.892 | -14523.16 |
| 1+NAO | 7 | 29060.58 | 5.39 | 0.067 | 0.929 | -14523.29 |
| 1+z.T4+z.P4 | 8 | 29061.11 | 5.92 | 0.052 | 0.959 | -14522.55 |
| 1 | 6 | 29061.87 | 6.68 | 0.035 | 0.979 | -14524.93 |
| 1+z.P4+NAO | 8 | 29062.57 | 7.38 | 0.025 | 0.993 | -14523.28 |
| 1+z.P4 | 7 | 29063.83 | 8.64 | 0.013 | 1 | -14524.92 |

Table S4.2. Model comparison for local and regional drivers influencing breeding success of the first clutch. 1 refers to the intercept, *z* refers to z-transformed values, *T* and *P* refer to temperature and precipitation, respectively, and the number behind them represents the month of the year. The number of parameters includes all control parameters and the residual standard deviation (Appendix S1).

| **model** | **number of parameters** | **DIC** | **ΔDIC** | **DIC weight** | **cumulative weight** | **log likelihood** |
| --- | --- | --- | --- | --- | --- | --- |
| 1+z.T5 | 8 | 12280.94 | 0 | 0.342 | 0.342 | -6139.701 |
| 1+z.T5+z.P5+z.T5:z.P5 | 10 | 12281.81 | 0.871 | 0.221 | 0.564 | -6139.064 |
| 1+z.T5+z.P5 | 9 | 12283.05 | 2.112 | 0.119 | 0.683 | -6139.517 |
| 1 | 7 | 12283.48 | 2.545 | 0.096 | 0.779 | -6139.177 |
| 1+z.T5+NAO | 9 | 12284.09 | 3.152 | 0.071 | 0.850 | -6138.459 |
| 1+z.P5 | 8 | 12285.14 | 4.203 | 0.042 | 0.892 | -6137.969 |
| 1+z.T5+z.P5+NAO | 10 | 12285.19 | 4.249 | 0.041 | 0.932 | -6140.574 |
| 1+z.T5+z.P5+NAO+z.T5:z.P5 | 11 | 12285.68 | 4.747 | 0.032 | 0.964 | -6140.761 |
| 1+z.P5+NAO | 9 | 12286.07 | 5.133 | 0.026 | 0.991 | -6141.625 |
| 1+NAO | 8 | 12288.13 | 7.193 | 0.009 | 1 | -6138.615 |

Table S4.3: Estimates of all test predictors affecting the timing of breeding and breeding success of first broods. T: temperature. P: precipitation. NAO: NAO index (see Methods and Appendix S1).

| Test predictor | Timing of Breeding | | | | Breeding success | | | |
| --- | --- | --- | --- | --- | --- | --- | --- | --- |
|  | Estimate | Standard error | p-value | Σ(ωAIC) | Estimate | Standard error | p-value | Σ(ωAIC) |
| T(April) | -1.2 | 0.59 | ^1^ | 0.92 | - | - | - | - |
| P(April) | -0.04 | 0.05 | ^1^ | 0.82 | - | - | - | - |
| T:P(April) | 0.08 | 0.03 | 0.01 | 0.71 | - | - | - | - |
| T(May) | - | - | - | - | 0.02 | 0.02 | ^1^ | 0.83 |
| P(May) | - | - | - | - | -0.0003 | 0.0005 | ^1^ | 0.48 |
| T:P(May) | - | - | - | - | -0.0001 | 0.0002 | 0.005 | 0.25 |
| NAO (linear) | -1.27 | 0.58 | 0.03 | 0.75 | 0.007 | 0.005 | 0.61 | 0.18 |

^1^ p-value not indicated because it is conditional on another predictor and thus does not have a meaningful interpretation (Aiken & West 1991; Schielzeth 2010)

## Second clutch

Table S4.4. Model comparison for local and regional drivers influencing the timing of breeding of the second clutch. 1 refers to the intercept. *z* refers to z-transformed values. *T* and *P* refer to temperature and precipitation, respectively, and the number behind them represents the month of the year. *poly(NAO, degree=2)* refers to the quadratic relation of the NAO index. The number of parameters includes all control parameters and the residual standard deviation (Appendix S1).

| **model** | **number of parameters** | **AIC** | **ΔAIC** | **AIC weight** | **cumulative weight** | **log likelihood** |
| --- | --- | --- | --- | --- | --- | --- |
| 1+z.T7+z.P7+poly(NAO, degree=2) +z.T7:z.P7 | 11 | 28055.33 | 0 | 0.42 | 0.42 | -14016.67 |
| 1+z.T7+z.P7+poly(NAO, degree=2) | 10 | 28055.52 | 0.19 | 0.38 | 0.81 | -14017.76 |
| 1+z.T7+poly(NAO, degree=2) | 9 | 28057.54 | 2.21 | 0.14 | 0.95 | -14019.77 |
| 1+z.P7+poly(NAO, degree=2) | 9 | 28060.16 | 4.83 | 0.04 | 0.99 | -14021.08 |
| 1+z.T7+z.P7+z.T7:z.P7 | 9 | 28063.7 | 8.36 | 0.01 | 0.99 | -14022.85 |
| 1+z.P7 | 7 | 28064.88 | 9.54 | 0 | 1 | -14025.44 |
| 1+z.T7+z.P7 | 8 | 28066.63 | 11.29 | 0 | 1 | -14025.31 |
| 1+z.T7 | 7 | 28067.18 | 11.84 | 0 | 1 | -14026.59 |
| 1 | 6 | 28067.65 | 12.32 | 0 | 1 | -14027.83 |
| 1+poly(NAO, degree=2) | 8 | 28067.81 | 12.47 | 0 | 1 | -14025.90 |

Table S4.5. Model comparison for local and regional drivers influencing breeding success of the second clutch. 1 refers to the intercept. *z* refers to z-transformed values. *T* and *P* refer to temperature and precipitation, respectively, and the number behind them represents the month of the year. *poly(NAO, degree=2)* refers to the quadratic relation of the NAO index. The number of parameters includes all control parameters and the residual standard deviation (Appendix S1).

| **model** | **number of parameters** | **DIC** | **ΔDIC** | **DIC weight** | **cumulative weight** | **log likelihood** |
| --- | --- | --- | --- | --- | --- | --- |
| 1+z.T8+z.P8+poly(NAO, degree=2) | 7 | 11313.19 | 0.00 | 0.748 | 0.748 | -5655.84 |
| 1+z.T8+z.P8+z.T8:z.P8 | 6 | 11317.13 | 3.94 | 0.104 | 0.853 | -5655.98 |
| 1+z.P8 | 4 | 11318.03 | 4.84 | 0.066 | 0.919 | -5657.92 |
| 1+z.T8+z.P8+poly(NAO, degree=2) +z.T8:z.P8 | 8 | 11318.55 | 5.37 | 0.051 | 0.970 | -5657.84 |
| 1+z.T8+z.P8 | 5 | 11321.13 | 7.94 | 0.014 | 0.984 | -5657.73 |
| 1+poly(NAO, degree=2) | 5 | 11322.37 | 9.18 | 0.008 | 0.992 | -5659.80 |
| 1+z.T8+poly(NAO, degree=2) | 6 | 11323.30 | 10.12 | 0.005 | 0.997 | -5658.00 |
| 1 | 3 | 11325.29 | 12.11 | 0.002 | 0.998 | -5658.90 |
| 1+z.T8 | 4 | 11325.55 | 12.36 | 0.002 | 1.000 | -5658.42 |
| 1+z.P8+poly(NAO, degree=2) | 6 | 11332.40 | 19.21 | 0.000 | 1.000 | -5660.13 |

Table S4.6: Estimates of all test predictors affecting the timing of breeding and breeding success of second broods. T: temperature. P: precipitation. NAO: NAO index (see Methods and Appendix S1).

| Test variable | Timing of Breeding | | | | Breeding success | | | |
| --- | --- | --- | --- | --- | --- | --- | --- | --- |
|  | Estimate | Standard error | p-value | Σ(ωAIC) | Estimate | Standard error | p-value | Σ(ωAIC) |
| T(July) | -1.73 | 0.95 | ^1^ | 0.96 | - | - | - | - |
| P(July) | -0.18 | 0.32 | ^1^ | 0.86 | - | - | - | - |
| T:P(July) | 0.03 | 0.02 | 0.1613 | 0.43 | - | - | - | - |
| T(August) | - | - | - | - | 0.008 | 0.002 | ^1^ | 0.92 |
| P(August) | - | - | - | - | -0.003 | 0.001 | ^1^ | 0.98 |
| T:P(August) | - | - | - | - | 0.0003 | 0.0001 | 0.05 | 0.16 |
| NAO (linear) | -16.46 | 25.79 | 0.4794 | 0.99 | 0.008 | 0.004 | <0.005 | 0.81 |
| NAO (quadratic) | 29.59 | 23.92 |  |  | 1.06 | 0.48 |  |  |
