# Supplementary material for: Earlier breeding, lower success: does the spatial scale of climatic conditions matter in a migratory passerine bird?
Source: Ecol Evol. 2015 Nov 19;5(23):5722–34. doi: 10.1002/ece3.1824 (PMC4813123; doi:10.1002/ece3.1824)
Supplement: Supplementary file 5 — Appendix S5 Estimates of random effects control predictors. [file ECE3-5-5722-s005.docx]

**Supporting Information 5. Estimates of random effects control predictors**

Local models always refer to models that investigate the effects of local temperature and precipitation, while regional models investigate the effects of NAO, exclusively. The Across-scale models include both, local weather and regional NAO. See Methods of the main text and Appendix S1 for more details.

Table S5.1. Estimates of random effects control predictors for LMMs of breeding timing for the first clutch. *Std. Dev.* refers to “Standard Deviation”.

| **Random effect** | **Local model** | | **Regional model** | | **Across-scale model** | |
| --- | --- | --- | --- | --- | --- | --- |
|  | **Variance** | **Std. Dev.** | **Variance** | **Std. Dev.** | **Variance** | **Std. Dev.** |
| Locality (intercept) | 10.63 | 3.26 | 6.19 | 2.49 | 10.97 | 3.31 |
| Year (intercept) | 6.77 | 2.60 | 11.04 | 3.32 | 5.93 | 2.44 |
| Latitude in year (slope) | 0.00 | 0.00 | 0.00 | 0.00 | 0.00 | 0.00 |

Table S5.2. Estimates of random effects control predictors for LMMs of breeding timing for the second clutch. *Std. Dev.* refers to “Standard Deviation”.

| **Random effect** | **Local model** | | **Regional model** | | **Across-scale model** | |
| --- | --- | --- | --- | --- | --- | --- |
|  | **Variance** | **Std. Dev.** | **Variance** | **Std. Dev.** | **Variance** | **Std. Dev.** |
| Locality (intercept) | 6.23 | 2.50 | 6.19 | 2.49 | 5.99 | 2.45 |
| Year (intercept) | 7.11 | 2.67 | 7.4e-6 | 2.7e-3 | 1.6e-3 | 0.04 |
| Latitude in year (slope) | 0.00 | 0.00 | 3.9e-3 | 0.06 | 5.8e-4 | 0.02 |

Table S5.3. Estimates of random effects control predictors for GLMMs of breeding success for the first clutch. *Std. Dev.* refers to “Standard Deviation”.

| **Random effect** | **Local model** | | **Regional model** | | **Across-scale model** | |
| --- | --- | --- | --- | --- | --- | --- |
|  | **Variance** | **Std. Dev.** | **Variance** | **Std. Dev.** | **Variance** | **Std. Dev.** |
| Locality (intercept) | 0.00 | 0.00 | 2.4e-14 | 1.6e-7 | 0.00 | 0.00 |
| Year (intercept) | 0.00 | 0.00 | 9.5e-14 | 3.1e-7 | 0.00 | 0.00 |
| Latitude in year (slope) | 0.00 | 0.00 | 6.18e-20 | 2.49e-10 | 0.00 | 0.00 |

Table S5.4. Estimates of random effects control predictors for GLMMs of breeding success for the second clutch. *Std. Dev.* refers to “Standard Deviation”.

| **Random effect** | **Local model** | | **Regional model** | | **Across-scale model** | |
| --- | --- | --- | --- | --- | --- | --- |
|  | **Variance** | **Std. Dev.** | **Variance** | **Std. Dev.** | **Variance** | **Std. Dev.** |
| Locality (intercept) | 0.00 | 0.00 | 5.2e-14 | 2.3e-7 | 0.00 | 0.00 |
| Year (intercept) | 5.72e-23 | 7.57e-12 | 7.0e-14 | 2.6e-7 | 0.08 | 0.29 |
| Latitude in year (slope) | 0.00 | 0.00 | 8.3e-21 | 9.1e-11 | 0.00 | 0.00 |
